# Role of Astrocytic Inwardly Rectifying Potassium (Kir) 4.1 Channels in Epileptogenesis

**DOI:** 10.3389/fneur.2020.626658

**Published:** 2020-12-23

**Authors:** Masato Kinboshi, Akio Ikeda, Yukihiro Ohno

**Affiliations:** ^1^Department of Pharmacology, Osaka University of Pharmaceutical Sciences, Takatsuki, Japan; ^2^Department of Epilepsy, Movement Disorders and Physiology, Graduate School of Medicine, Kyoto University, Kyoto, Japan

**Keywords:** astrocytes, Kir4.1 channels, spatial potassium buffering, epilepsy, BDNF, glutamate, tripartite synapse

## Abstract

Astrocytes regulate potassium and glutamate homeostasis via inwardly rectifying potassium (Kir) 4.1 channels in synapses, maintaining normal neural excitability. Numerous studies have shown that dysfunction of astrocytic Kir4.1 channels is involved in epileptogenesis in humans and animal models of epilepsy. Specifically, Kir4.1 channel inhibition by *KCNJ10* gene mutation or expressional down-regulation increases the extracellular levels of potassium ions and glutamate in synapses and causes hyperexcitation of neurons. Moreover, recent investigations demonstrated that inhibition of Kir4.1 channels facilitates the expression of brain-derived neurotrophic factor (BDNF), an important modulator of epileptogenesis, in astrocytes. In this review, we summarize the current understanding on the role of astrocytic Kir4.1 channels in epileptogenesis, with a focus on functional and expressional changes in Kir4.1 channels and their regulation of BDNF secretion. We also discuss the potential of Kir4.1 channels as a therapeutic target for the prevention of epilepsy.

## Introduction

Epilepsy is a common neurological disease that is characterized by recurrent seizures caused by neuronal hyperexcitation. The current antiepileptic agents predominantly act on neuronal ion channels (e.g., blockers of voltage-gated sodium channels and calcium channels), glutamate receptors [e.g., antagonists of a-amino-3-hydroxy-5-methyl-isoxazolopropionic acid (AMPA) receptors], or GABAergic inhibitory systems (e.g., modulators of the GABA_A_ receptor/chloride channel complex and inhibitors of GABA transaminase), which aim to suppress excessive neural excitation (1, 2). Therapy with these antiepileptic drugs is effective in about 70% of epilepsy patients, whereas seizure control is not achieved for the remaining 30% of patients (3, 4). Thus, there is a high unmet need for novel therapeutic targets or agents to treat refractory epilepsy.

Numerous findings show that astrocytes, the major cell component of glial cells in the central nervous system (CNS), actively regulate the excitability and plasticity of neurons by forming tripartite synapses in conjunction with presynaptic and postsynaptic neural components (5–10). Specifically, astrocytes regulate ion homeostasis and extracellular space volume, metabolize neurotransmitters (e.g., glutamate, GABA, and glycine), and secrete various neuroactive molecules including gliotransmitters [e.g., glutamate, D-serine, adenosine 5′-triphosphate (ATP)], neurotrophic factors [e.g., brain-derived neurotrophic factor (BDNF) and glia-derived neurotrophic factor (GDNF)], and cytokines [e.g., tumor necrosis factor-α (TNF-α) and interleukin-1β (IL-1β)] (11–13). Among these functions of astrocytes, a spatial buffering system for potassium ions (K^+^) plays an important role in the maintenance of neuronal excitability, which transports excessive extracellular K^+^ secreted from excited neurons to sites with lower K^+^ concentrations (e.g., microcapillaries) (14–18). This potassium clearance mechanism is primarily mediated by astrocytic inwardly rectifying potassium (Kir) channels containing Kir4.1 subunits (Kir4.1 channels) (16–21).

In this review, we introduce the current understanding regarding the pathophysiological role of astrocytic Kir4.1 channels in the development of epilepsy (epileptogenesis). Further, we discuss the potential of Kir4.1 channels as a therapeutic target for the prevention of epilepsy.

## Spatial Potassium Buffering and Astrocytic Kir4.1 Channels

**Figure 1:**
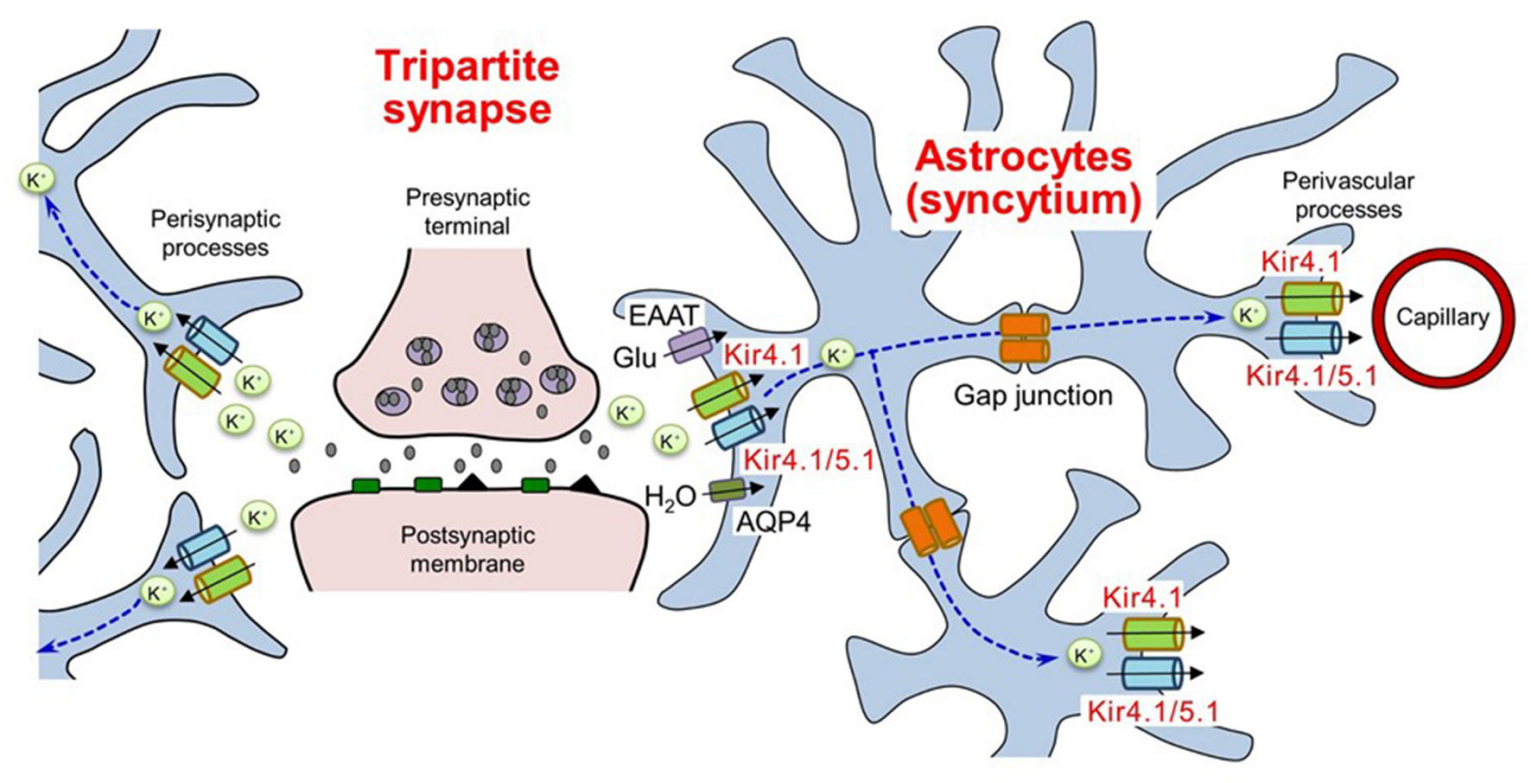
Spatial K^+^ buffering of astrocytes in tripartite synapses. Astrocytes uptake extracellular K^+^ secreted from neurons and release K^+^ in regions with lower K^+^ levels by coupling into a syncytium through gap junctions. The K^+^ buffering mechanism is corelated with glutamate uptake and water transport by astrocytes. EAAT, excitatory amino acid transporters; AQP4, aquaporin 4. Modified from Ohno et al. (25).

Extracellular K^+^ levels are critical for determining the resting membrane potential of neurons and are normally maintained at ca. 3–5 mM (22). Physiological neural activity leads to an elevation of <1 mM in extracellular K^+^ concentration (23). Increases of 10–12 mM K^+^ in ceiling levels are induced during excessive neural activity due to electrical stimulation (24). Astrocytes rapidly transport K^+^ from synapses, where K^+^ is secreted from neurons during the repolarization phase, to regions with lower K^+^ levels (e.g., microcapillaries) by coupling into a syncytium through gap junctions (Figure 1) (14–21, 25). This astrocytic K^+^ clearance mechanism, known as “spatial potassium buffering,” is vital for maintaining K^+^ homeostasis and preventing neural hyperexcitability during normal brain function. In addition, spatial potassium buffering is known to be linked to glutamate uptake via glutamate transporters [e.g., excitatory amino acid transporters 1 (EAAT1) and EAAT2] and water transport via aquaporin-4 (AQP4) by astrocytes (26–31). Moreover, both connexin30 and connexin43 in astrocytic gap junctions were shown to play a critical role in normal K^+^ redistribution, using double knockout techniques in mice (32, 33).

**Figure 2:**
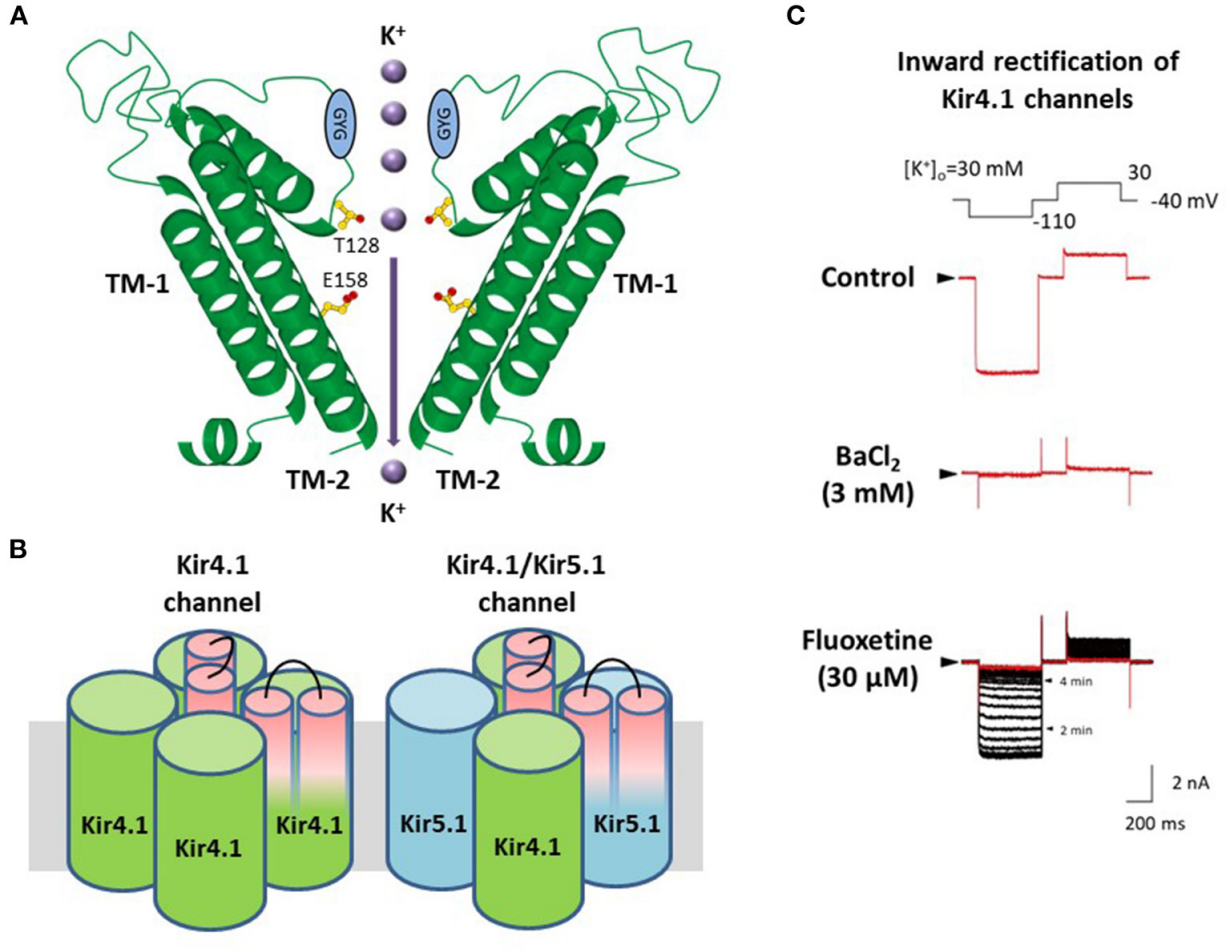
Molecular structure and properties of Kir4.1 channels. **(A)** Kir4.1 subunits have two transmembrane (TM) helices with one extracellular loop, including the GYG signature sequence of the K^+^ selectivity filter. **(B)** Kir4.1 subunits construct two types of channels, the homo-tetramer of Kir4.1 and the hetero-tetramer of Kir4.1 and Kir5.1. **(C)** Kir4.1 channels (homo-tetramer of Kir4.1) conduct large inward and relatively small outward K^+^ currents. Selective serotonin reuptake inhibitors, fluoxetine, inhibit Kir4.1 channel currents. Modified from Ohno et al. (25).

The influx of K^+^ into astrocytes is mainly mediated by Kir channels containing Kir4.1 and Kir5.1 subunits, which are highly expressed in astrocytes and retinal Müller cells (16–21, 34–39). Kir4.1 subunits have two transmembrane (TM) domains with an extracellular ion selectivity filter, including the GYG signature sequence, which construct Kir channels by forming tetramers (Figure 2) (25, 39). Two types of Kir4.1-containing Kir channels (Kir4.1 channels), the homo-tetramer of Kir4.1 and the hetero-tetramer of Kir4.1 and Kir5.1, conduct large inward K^+^ currents at potentials negative to K^+^ equilibrium potential (E_K_) and moderate outward K^+^ currents at those positive to E_K_ (Figure 2) (25, 39, 40).

Pharmacological studies have shown that among CNS agents, several antidepressants reversibly inhibited K^+^ currents via Kir4.1 channels in a subunit-dependent manner. Tricyclic antidepressants (TCAs) such as nortriptyline, amitriptyline, desipramine, and imipramine, blocked Kir4.1 channels in a voltage-dependent manner, while selective serotonin reuptake inhibitors (SSRIs) including fluoxetine and sertraline inhibited Kir4.1 channels in a voltage-independent manner (Figure 2) (41–44). The inhibitory effects of antidepressants for Kir4.1 channels were achieved at concentrations considered to be within a range of brain concentrations for clinical treatment of depression. Antidepressant treatment is reported to increase the risk of seizure incidence (45, 46), which may be due to antidepressant drug actions on Kir4.1 channels.

Alanine-scanning mutagenesis studies on the antidepressant-Kir4.1 channel interaction demonstrated that these antidepressant agents specifically blocked the Kir4.1 channel pore (47). Two amino acids, T128 and E158, on pore and TM-2 helices respectively, can bind to antidepressants. Recently, anti-malarial agents such as quinacrine and chloroquine, and the anti-protozoal agent, pentamidine, have also been shown to inhibit Kir4.1 channels by binding to T128 and E158, similar to antidepressant agents (48–50). Although few reports are available on drug-Kir4.1 interaction, information about structure-based action on Kir4.1 channels is important for designing novel treatment compounds for epilepsy and reducing the potential of seizure side effects.

## Kir4.1 Channels in Epilepsy Patients

Mutations in the human *KCNJ10* gene encoding Kir4.1 were reported to cause the epileptic disorders known as “EAST” (Epilepsy, Ataxia, Sensorineural deafness and Tubulopathy) and “SeSAME” (Seizures, Sensorineural deafness, Ataxia, Mental retardation, and Electrolyte imbalance) syndrome (OMIM 612780) (51–53). Patients with EAST/SeSAME syndrome initially manifest generalized tonic-clonic seizures (GTCSs) within a few months after birth and are treated with anticonvulsant agents such as valproate and phenobarbital. The *KCNJ10* mutations responsible for EAST/SeSAME syndrome have been shown to be T57I, R65P, R65C (cytoplasmic end of TM-1), F75L, F75C, G77R (TM-1), V91Gfs^*^197, F119Gfs^*^25, C140R (extracellular loop between TM-1 and TM-2), T164I, A167V (TM-2), R175Q, R199X, R240H, V259^*^, G275Vfs^*^7, and R297C (C-terminal domain) (51–64). These homozygous or compound heterozygous mutations disrupted Kir4.1 channel function to varying degrees from completely to moderately. Moreover, novel loss-of-function mutations (I60T, I60M, G163D, R171Q, A201T, I209T, and T290A) in *KCNJ10* were identified in patients with atypical EAST/SeSAME syndrome lacking one or more core clinical manifestations (65–69). In contrast, heterozygous gain-of-function mutations (R18Q, V84M, and R348H) in *KCNJ10* caused autism spectrum disorders with spastic seizures and intellectual disability (70–72).

Electrophysiological investigations demonstrated that Kir currents were significantly reduced in hippocampal specimens from refractory temporal lobe epilepsy (TLE) patients, using patch-clamp techniques (73–75). The impairment of glial K^+^ uptake sensitive to Ba^2+^, a blocker of Kir channels, was also in sclerotic hippocampal slices from patients with epilepsy (76, 77). Furthermore, astrocytic Kir4.1 expression has been shown to decrease in the sclerotic hippocampus of TLE patients (78–80). Additionally, flavoprotein fluorescence imaging, visualizing neuronal activities without exogenous dyes in living tissues taken from epilepsy patients, showed that epileptiform activities propagated from the subiculum of the hippocampus with sclerosis, where the Kir4.1 expression of astrocytes was markedly down-regulated (81). In refractory partial epilepsy pathologically diagnosed as “focal cortical dysplasia type 1,” Kir4.1 expression was decreased in the epileptogenic regions where direct current (DC) shifts were detected using wide-band electroencephalography (EEG) recordings (82). These DC shifts preceding conventional ictal pattern and high frequency oscillations (HFOs), known as “active DC shifts,” were suggested to reflect the extracellular K^+^ accumulation caused by the dysfunction of astrocytic potassium buffering, which can be EEG biomarkers for the epileptic zone (82). Therefore, Kir4.1 channel dysfunction affected by gene mutations or expressional down-regulation is likely to be involved in the pathogenesis of human epileptic disorders.

## Kir4.1 Channels in Animal Epilepsy Models

Kir4.1 homozygous deletion in mice reduced body weight gain and caused progressive weakness by postnatal day (P) 8–10, although heterozygous mice showed no pathological behavior (83, 84). Subsequently, Kir4.1 knockout mice exhibited jerky movements and severe deficits in controlling voluntary movements, posture, and balance, and consequently died by P24. In addition, studies using conditional knockout techniques have reported that mice with conditional knockout of astrocytic Kir4.1 developed pronounced body tremor, ataxia, and stress-induced GTCSs, which were suggested to be involved in astrocytic membrane depolarization and impaired uptake of extracellular K^+^ following neural activity (Table 1) (30, 85, 86). Moreover, Kir4.1 conditional knockout also reduced glutamate uptake by astrocytes (30). This impairment of glutamate clearance resulted from the dysfunction of EAATs due to membrane depolarization in astrocytes (29–31, 87, 88).

**Table 1:** Pathophysiological changes in Kir4.1 channels in animal epilepsy models.

**Animal model**	**Functional and expressional changes in Kir4.1**	**Pathological behaviors and seizure types**
Conditional knockout mice of astrocytic Kir4.1	Dysfunction of Kir4.1 channel Impaired uptake of extracellular K^+^ and glutamate	Body tremor, ataxia, stress-induced GTCSs, premature death
Noda epileptic rats (NER)	Down-regulation of Kir4.1 expression in the amygdala	Spontaneous GTCSs
*Lgi1* mutant rats (ADLTE model)	Down-regulation of Kir4.1 expression in the temporal lobe after development of audiogenic epilepsy	Audio-induced GTCSs
Seizure susceptible DBA/2 mice	*Kcnj10* SNP with T262S variation Dysfunction of Kir4.1 channel Impaired uptake of glutamate	Increased seizure susceptibility
Trauma-induced epilepsy rats	Down-regulation of Kir4.1 expression in the cerebral cortex	Spontaneous partial seizures of cerebral cortex origin
Albumin-induced epilepsy rats	Down-regulation of Kir4.1 expression in the hippocampus exposed to albumin	Increased seizure susceptibility due to hippocampal hyperexcitability
Electrical stimulation-induced TLE rats	Transient reduction of Kir4.1 expression in the temporal cortex 24 hours after SE	No assessment
Groggy rats (absence epilepsy model)	No change in Kir4.1 expression	Absence-like seizures, ataxia
db/db mice (type 2 diabetic model)	Down-regulation of Kir4.1 expression in the hippocampus	Hippocampal hyperexcitability

Numerous studies using animal models of epilepsy showed that astrocytic Kir4.1 expressional changes were involved in seizure induction and susceptibility. Specifically, Kir4.1 expression was significantly reduced in Noda epileptic rats (NER), a hereditary epilepsy model (Table 1) (89). NER exhibited frequent spontaneous GTCSs associated with two genetic loci, chromosome (Chr) 1q32-33 and Chr5q22, including *cholecystokinin B receptor (Cckbr), suppressor of tumorigenicity 5 (St5)*, and *PHD finger protein 24 (Phf24)* (90–95). In NER, Kir4.1 expression was region-specifically reduced in the amygdala, where the expression of Fos protein, a biological marker of neural excitation, significantly elevated (89). Moreover, *Leucine-Rich Glioma-Inactivated 1 (Lgi1)* mutant rats, a model of human autosomal dominant lateral temporal lobe epilepsy (ADLTE), showed reduced astrocytic Kir4.1 expression in specific regions, including both the lateral and medial temporal lobes, after the acquisition of audiogenic seizure susceptibility (Table 1) (96). In these regions, neural hyperexcitation during seizures was confirmed using Fos immunohistochemical techniques (97). Auditory stimuli for seizure induction consisted of sound stimulation twice, priming stimulation at P16 and test stimulation at 8 weeks. Priming stimulation induces epileptogenesis caused by *Lgi1* mutation without spontaneous seizure phenotypes (96, 98). Interestingly, the Kir4.1 expression in astrocytes was reduced during the time-course of epileptogenesis before application of test stimulation at the age of 8 weeks in *Lgi1* mutant rats (96). These findings indicate that the dysfunction of Kir4.1 channels is involved not only in evoking seizure generation, but also in chronic development of epilepsy (epileptogenesis).

Furthermore, the *Kcnj10* single nucleotide polymorphism (SNP) with T262S variation that disrupts Kir4.1 channel activity has been identified as the mutation responsible for seizure susceptibility of DBA/2 mice (Table 1) (99, 100). A rodent epilepsy model induced by fluid percussion injury or albumin injection also exhibited down-regulation of Kir4.1 expression in regions related to seizure foci (Table 1) (101, 102). Kir4.1 expression was transiently reduced after status epilepticus (SE) in temporal lobe epilepsy (TLE) models induced by electrical stimulation, although the expression of Kir4.1 returned to the normal level 1 week after SE (Table 1) (103). In contrast to epilepsy models with convulsive seizures, no changes in Kir4.1 expression were detected in Groggy rats, an absence epilepsy model (Table 1) (104). In addition, hyperglycemia has been reported to reduce Kir4.1 expression and disrupt the clearance of both K^+^ and glutamate using astrocyte primary cultures (105). Type 2 diabetic mice (db/db) also showed down-regulation of Kir4.1 expression and dysfunction in K^+^ intake that were associated with hippocampal neural hyperexcitability (Table 1) (106). These studies may explain the epileptic predisposition of type 2 diabetes patients (107, 108).

## Astrocytic Kir4.1-BDNF System in Epileptogenesis

BDNF is a member of the neurotrophin family essential for the normal development and function of the CNS. Specifically, BDNF regulates cell survival, neurogenesis, neuronal sprouting, synaptic plasticity, and reactive gliosis by binding to tropomyosin-related kinase (Trk) receptors, especially TrkB receptors (109–112). The neurotrophic properties of BDNF potentially produce therapeutic effects for neurodegenerative diseases (e.g., Alzheimer's disease, Parkinson's disease, and Huntington's disease) and neuropsychiatric diseases (e.g., depression and bipolar disorder) (113–119). However, elevated expression of BDNF is known to be involved in the pathogenesis of epilepsy in various animal models and human brains (111, 120, 121). In addition, inhibition of BDNF/TrkB signaling has been shown to suppress the development of epilepsy in animal models (122–126).

While the expressional levels of BDNF were higher in neurons, astrocytic BDNF expression and BDNF/TrkB signaling have also been shown to contribute to brain functions under physiological and pathophysiological conditions (127–130). A recent study demonstrated that BDNF overexpression in astrocytes caused neuronal hyperexcitability and cell death, and deteriorated the phenotypes in lithium pilocarpine-induced TLE models, which were suggested to be mediated by astrocytic TrkB receptors, rather than neural TrkB receptors (131).

**Figure 3:**
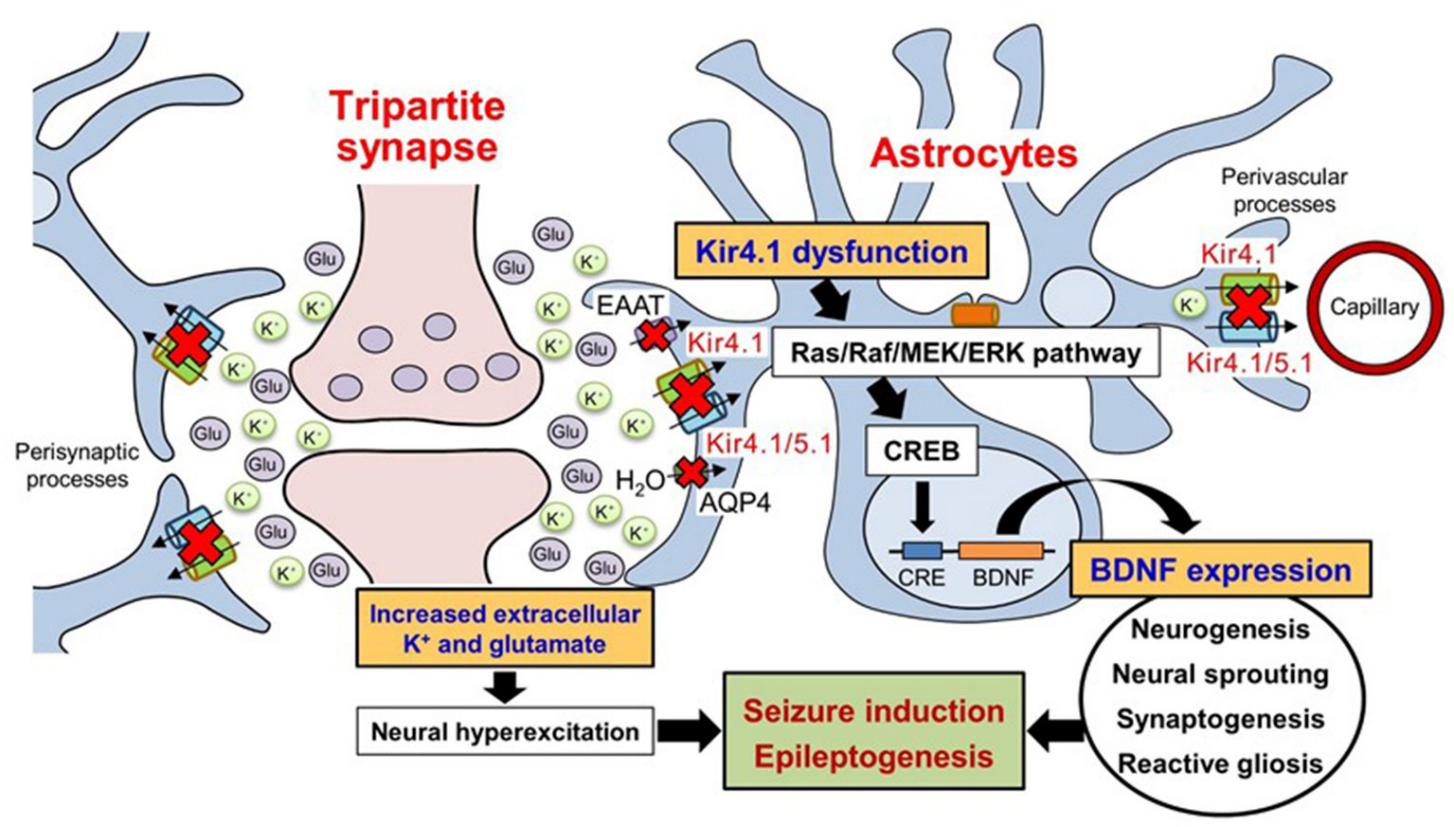
Schematic drawing illustrating the effects of Kir4.1 dysfunction on neural hyperexcitation and astrocytic BDNF expression. Dysfunction (genetic mutation, down-regulated expression, and pharmacological blockade) of Kir4.1 channels increases extracellular K^+^ and glutamate at synapses and causes neural hyperexcitability. The dysfunction of Kir4.1 channels activates the Ras/Raf/MEK/ERK signaling pathway and facilitates BDNF expression in astrocytes. Based on these changes, astrocytic Kir4.1 channels play important roles in modulating seizure induction and epileptogenesis. Modified from Ohno et al. (25).

Astrocytic Kir4.1 channels have been shown to modulate BDNF expression using astrocyte primary cultures (25, 44, 132). Several antidepressant agents (e.g., imipramine and amitriptyline), which reportedly inhibited Kir4.1 channels in a subunit-specific manner (41, 43, 47), facilitated the expression of BDNF in astrocytes (133–135). Furthermore, the relative potencies of antidepressant agents for BDNF induction were consistent with those for the blockade of Kir4.1 channels, but not for the inhibition of 5-HT reuptake (43, 44). In addition, Kir4.1 knockdown by small interfering RNA (siRNA) transfection significantly elevated BDNF expression in astrocytes, which was suppressed by a MEK1/2 inhibitor, but not by a p38 MAPK inhibitor or a JNK inhibitor (44). These results suggest that the reduced function of Kir4.1 channels facilitates BDNF expression in astrocytes by activating the Ras/Raf/MEK/ERK pathway (Figure 3) (25, 44, 132). This hypothesis was supported by previous studies showing that the Ras/Raf/MEK/ERK signaling pathway regulates the transcription of BDNF and other survival/plasticity genes through interaction with cyclic AMP response element binding protein (CREB) (136, 137). It is therefore likely that Kir4.1 channels play a key role in modulating epileptogenesis by controlling not only the extracellular K^+^ and glutamate levels in synapses, but also the BDNF expression in astrocytes. The astrocytic Kir4.1-BDNF system is expected to serve as a novel target for the treatment of epilepsy, especially epileptogenesis.

## Kir4.1 Channels as a Novel Therapeutic Target for Prevention of Epilepsy

Based on the potential role of astrocytic Kir4.1 channels in epileptogenesis, normalizing the down-regulation of astrocytic Kir4.1 channel expression during epileptogenesis can be a therapeutic strategy to prevent epilepsy. We recently showed that repeated treatments with antiepileptic drugs (valproate, phenytoin, and phenobarbital), which are effective for convulsive seizures, commonly elevated the astrocytic Kir4.1 expression in the limbic region (138). These antiepileptic drugs have previously showed inhibitory effects on kindling development in animal models (139–141), in which the elevated expression of Kir4.1 channels may contribute to the prophylactic effect of these drugs. Moreover, we have shown that valproate prevented audiogenic epileptogenesis in *Lgi1* mutant rats by elevating the down-regulated Kir4.1 expression during epileptogenesis in a dose-dependent manner (96). Although further studies are required to reveal the mechanisms underlying the Kir4.1 pathogenic changes in epileptogenesis, these findings support the notion that astrocytic Kir4.1 channels can be therapeutic targets for prevention of epilepsy. Specifically, novel compounds positively modulating astrocytic Kir4.1 channels are expected to have potential for treatment of epilepsy and epileptogenesis. Although no information on the structure-activity relationship for Kir4.1 channel stimulators is available, gain-of-function mutations of the *KCNJ10* gene (e.g., R18Q in N-terminus and V84M in TM1 region) reported in patients with autism spectrum disorders may give hints for new drug discovery (70, 72). In addition, retigabine (ezogabine), an antiepileptic drug for focal onset seizures, may also give information since it primarily acts on neural KCNQ2-5 (K_v_7.2-7.5) ion channels as a positive allosteric modulator (142).

## Conclusion

Astrocytic Kir4.1 channels play a critical role in the regulation of brain homeostasis and neural excitability. Evidence is accumulating that dysfunction of astrocytic Kir4.1 channels is involved in epileptogenesis in both epilepsy patients and animal epilepsy models. Moreover, the reduced activity of Kir4.1 channels elevates the levels of extracellular K^+^ and glutamate at tripartite synapses and facilitates astrocytic BDNF expression, which can promote the development of epilepsy. Although data are limited, the approach to restore Kir4.1 down-regulation during epileptogenesis was actually effective to prevent the development of epilepsy in an animal model of epilepsy. Thus, the Kir4.1-BDNF system in astrocytes is expected to serve as a novel therapeutic target for epilepsy. especially epileptogenesis.

## Author Contributions

MK, AI, and YO designed and wrote the manuscript. All authors contributed to the article and approved the submitted version.

## Conflict of Interest

AI and MK belong to Department of Epilepsy, Movement Disorders and Physiology at Kyoto University is Industry-Academia Collaboration Courses, supported by Eisai Co., Ltd., Nihon Kohden Corporation, Otsuka Pharmaceutical Co., and UCB Japan Co., Ltd. The remaining author declares that the research was conducted in the absence of any commercial or financial relationships that could be construed as a potential conflict of interest.
